# Hydrogel-encapsulated extracellular vesicles for the regeneration of spinal cord injury

**DOI:** 10.3389/fnins.2023.1309172

**Published:** 2023-12-14

**Authors:** Yasaman Nazerian, Amirhossein Nazerian, Fereshteh Mohamadi-Jahani, Parastoo Sodeifi, Maryam Jafarian, Seyed Amir Hossein Javadi

**Affiliations:** ^1^Brain and Spinal Cord Injury Research Center, Neuroscience Institute, Tehran University of Medical Sciences, Tehran, Iran; ^2^School of Medicine, Iran University of Medical Sciences, Tehran, Iran; ^3^School of Medicine, Islamic Azad University of Medical Sciences, Tehran, Iran; ^4^Department of Neurosurgery, Imam Khomeini Hospital Complex, Tehran University of Medical Sciences, Tehran, Iran

**Keywords:** spinal cord injuries, stem cells, cell transplantation, EVS, hydrogel, nerve repair

## Abstract

Spinal cord injury (SCI) is a critical neurological condition that may impair motor, sensory, and autonomous functions. At the cellular level, inflammation, impairment of axonal regeneration, and neuronal death are responsible for SCI-related complications. Regarding the high mortality and morbidity rates associated with SCI, there is a need for effective treatment. Despite advances in SCI repair, an optimal treatment for complete recovery after SCI has not been found so far. Therefore, an effective strategy is needed to promote neuronal regeneration and repair after SCI. In recent years, regenerative treatments have become a potential option for achieving improved functional recovery after SCI by promoting the growth of new neurons, protecting surviving neurons, and preventing additional damage to the spinal cord. Transplantation of cells and cells-derived extracellular vesicles (EVs) can be effective for SCI recovery. However, there are some limitations and challenges related to cell-based strategies. Ethical concerns and limited efficacy due to the low survival rate, immune rejection, and tumor formation are limitations of cell-based therapies. Using EVs is a helpful strategy to overcome these limitations. It should be considered that short half-life, poor accumulation, rapid clearance, and difficulty in targeting specific tissues are limitations of EVs-based therapies. Hydrogel-encapsulated exosomes have overcome these limitations by enhancing the efficacy of exosomes through maintaining their bioactivity, protecting EVs from rapid clearance, and facilitating the sustained release of EVs at the target site. These hydrogel-encapsulated EVs can promote neuroregeneration through improving functional recovery, reducing inflammation, and enhancing neuronal regeneration after SCI. This review aims to provide an overview of the current research status, challenges, and future clinical opportunities of hydrogel-encapsulated EVs in the treatment of SCI.

## 1 Introduction

Spinal cord injury (SCI) is the most critical complication of spinal injury. The overall global incidence of SCI was 0.9 million cases and 12 cases per 100,000 persons in 2019 (Wang et al., 2022). Most of surviving patients with traumatic SCI suffer from SCI-related complications. Primary injury consists of neuronal and glial cell damage, neuroinflammation, and impairment of axonal regeneration, which are caused by the initial compression or contusion of the spinal cord. Excessive inflammatory responses, vascular network damage, and disruption of the blood-spinal cord barrier (BSCB) following mechanical injury initiate a secondary injury cascade. Secondary injury cascade consists of hypoperfusion, ischemia, oxidative damages, and inflammatory changes leading to progressive spinal cord dysfunction and consequent sensory, motor, and autonomic function loss (Hellenbrand et al., 2021; Michel et al., 2021). Furthermore, SCI causes short-term or long-term dysfunctions in multiple organs such as the respiratory system, reproductive system, cardiovascular system, digestive system, urogenital system, and skeletal system (Sweis and Biller, 2017). These complications can significantly impact a person’s quality of life, increase hospitalization rates, prolong recovery times, and even lead to death. Acute phase management and restraining the secondary injury is largely determining the final nerve recovery outcomes. However, there are currently no effective treatments for complete recovery after SCI (Flack et al., 2022). The blood–brain barrier (BBB) and BSCB are in fact common barriers that pharmacological strategies for neuro-oncology and neuro-traumatology have to bypass. Therefore, encapsulating drugs into biocompatible and biodegradable vectors might be a valid delivery strategy. Hyaluronic acid could be a valuable example which was suggested for similar purposes in the diagnosis and treatment of high-grade gliomas. Considering the wider range of hydrogels materials overcomes specific limitations of hyaluronic acid because other materials could offer more options in terms of mechanical strength, size and degradation rates (Ganau, 2014; Ganau et al., 2017). Currently available treatments mainly rely on suppressing damage progression, rehabilitation, prevention of complications, and other conservative approaches, which are limited in their effectiveness. Therefore, an effective strategy is critical in promoting neuronal regeneration and repairing SCI (Ahuja et al., 2017). Treatment strategies for better restoration of neurological function after spinal cord injury should be focused on minimizing the primary injury and preventing secondary injury, including attenuating inflammatory and oxidative responses, promoting remyelination, enhancing regeneration of axons, and promoting angiogenesis (Ahuja et al., 2017).

In recent years, regenerative treatments have become a potential option for the treatment of pathological brain conditions such as cancer, stroke, neurodegenerative diseases, and SCI (Burns and Quinones-Hinojosa, 2021; Ghasempour et al., 2022). These regenerative strategies have shown potential in achieving improved functional recovery after SCI by promoting the growth of new neurons, protecting surviving neurons, and preventing additional damage to the spinal cord. This leads to regeneration and repair of damaged tissue and restoration of lost functions (Ashammakhi et al., 2019). Cell transplantation as a regenerative therapy can be effective for SCI recovery through immunomodulation, enhancing angiogenesis, and promoting axonal regeneration and reinnervation (Hall et al., 2022). Currently, many cell-based clinical trials are being conducted to promote neuroregenerative (Donovan and Kirshblum, 2018). Although many types of cells exhibited promising functional recovery after SCI, the direct application of cells to target sites is still limited in the clinic. Different types of cells, such as embryonic stem cells, induced pluripotent stem cells (iPSC), neural precursor cells (NPCs), mesenchymal stem cells (MSCs), Schwann cells (SCs), and olfactory ensheathing cells (OECs) have been evaluated to promote tissue repair after SCI (Hejrati and Fehlings, 2021). Direct transplantation of some types of these cells may have some limitations such as safety and ethical concerns and limited efficacy due to the low survival rate of transplanted cells, immune rejection, and tumor formation (Wang et al., 2021). However, some of these challenges of cell-based therapy can be overcome (Hejrati et al., 2023). Using extracellular vesicles (EVs) derived from different types of cells is one of the helpful strategies to overcome these limitations without rising cell therapy concerns due to their lower immunogenicity and cytotoxicity and better bioavailability and biocompatibility (Zeng et al., 2022; Amirzadeh gougheri et al., 2023; Moeinabadi-Bidgoli et al., 2023).

**Figure 1:**
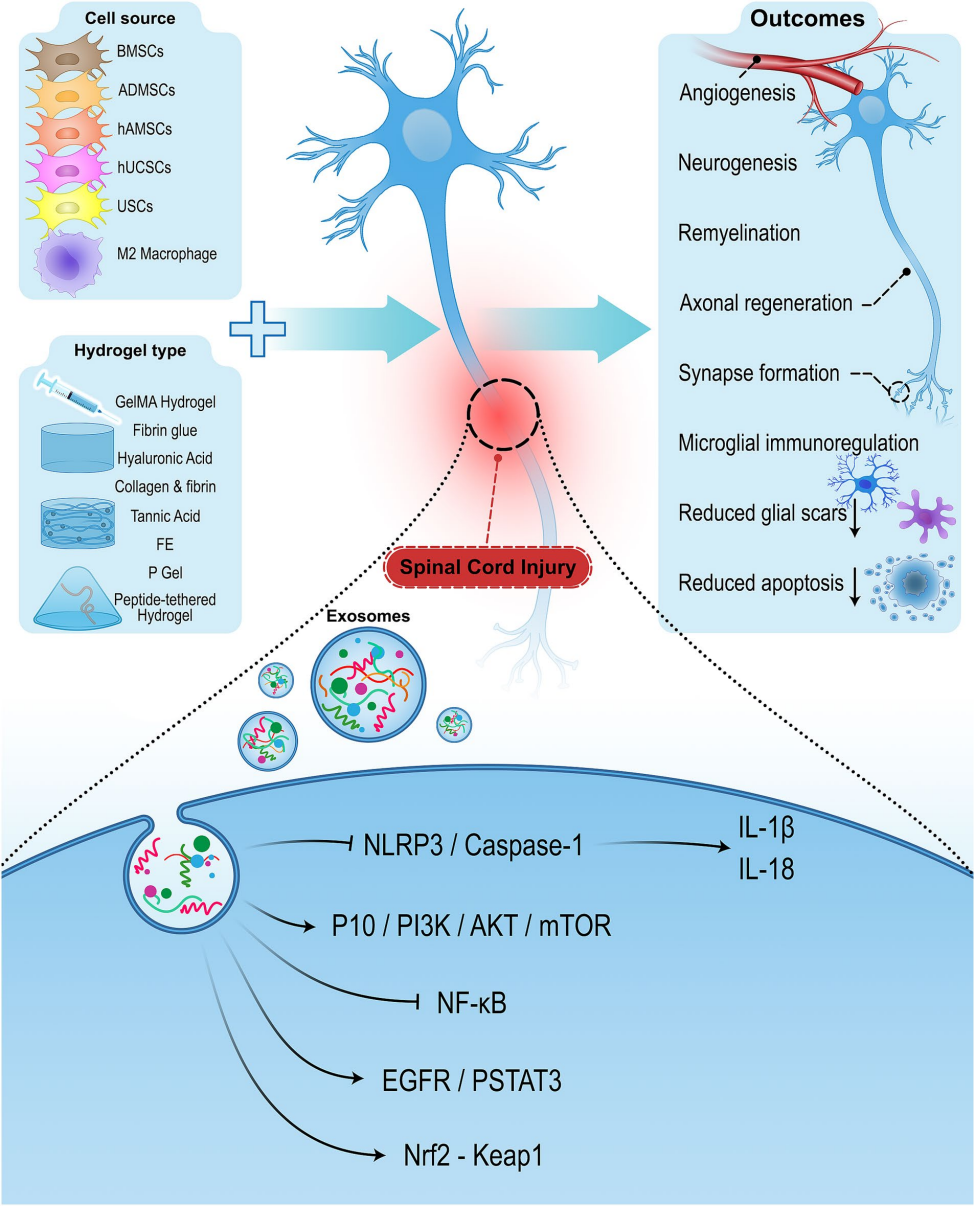
Schematic diagram of extracellular vesicles combined with hydrogels for the regeneration of SCI. Using hydrogel-encapsulated extracellular vesicles promotes neurological recovery by enhancing angiogenesis, neurogenesis, remyelination, axonal regeneration, synapse formation, and immunoregulation and reducing glial scars and apoptosis.

EVs specially exosomes, exert therapeutic impacts in the repair of SCI as they have the capability of neuroprotection, anti-inflammation, low immunogenicity, neuronal regeneration, and easy transportation. They can also attenuate the process of secondary injury and its co-morbidities (Huang et al., 2017; Sun et al., 2018; Dutta et al., 2021; Romanelli et al., 2021). Nevertheless, EV-based strategies in SCI treatment also have some limitations. Conventional EV-based strategies that rely on local repeated injection and systemic administrations result in low efficiency due to short half-time, poor accumulation, rapid clearance, and difficulty in targeting specific tissues (Imai et al., 2015; Lankford et al., 2018). The proper functioning of exosomes is dependent on their integration into the injured spinal cord tissue. On the other hand, the recovery process of the spinal cord is complex and lengthy, and EVs need to be retained in the injured site for an extended time. So, combining EVs with a vehicle that can serve as a sustained release carrier for EVs can exhibit efficient retention at the site of injury (Riau et al., 2019; Wang et al., 2021). There is growing interest in the use of hydrogel-encapsulated EVs to address these challenges. The combination of hydrogels and cell-derived EVs can be a promising strategy to enhance the efficacy of exosomes by maintaining their bioactivity, protecting EVs from rapid clearance, and facilitating the sustained release of EVs at the target site (Riau et al., 2019). Encapsulating exosomes with hydrogels have been applied in several fields and diseases, such as bone and cartilage regeneration, wound repair, corneal damage, myocardial infarction, and SCI (Riau et al., 2019; Xie et al., 2022; Zhou et al., 2022). Preclinical studies have shown that hydrogel-encapsulated EVs can improve functional recovery, reduce inflammation, and enhance neuronal regeneration in SCI animal models (illustrated in Figure 1). The goal of this review is to provide an overview of the current research status of hydrogel-encapsulated cell-derived EVs in neuroprotection and SCI functional recovery with a focus on describing their underlying mechanisms of action. The challenges and opportunities for future clinical applications are also discussed.

## 2 Pathophysiological mechanisms following spinal cord injury

Principal correlated destructive events that contribute to SCI pathogenesis are ischemia, oxidative stress, inflammatory responses, and apoptotic pathways. The primary injury phase consists of principal pathologic events such as destruction of neural parenchyma, disruption of axonal network, hemorrhage, and disruption of glial membrane. The secondary injury, that is triggered by primary injury, promotes permeability and vascular alterations, vasogenic edema, glutamate excitotoxicity, metabolic alterations, impaired inflammatory responses, axonal degeneration, demyelination, and fibroglial scarring initiation (Anjum et al., 2020; Lima et al., 2022). Trauma-induced neuroplastic processes cause white and gray matter atrophy and microstructural disrupted integrity. Spinal cord microstructural disruption in SCI patients are likely caused by changes in myelin, axonal density, and iron deposition, metabolic alterations, and ECM disruption. These microstructural disruptions may be associated with sensory/motor impairments and may limit the therapeutic efficacy (Ziegler et al., 2018; Guo et al., 2019).

**Figure 2:**
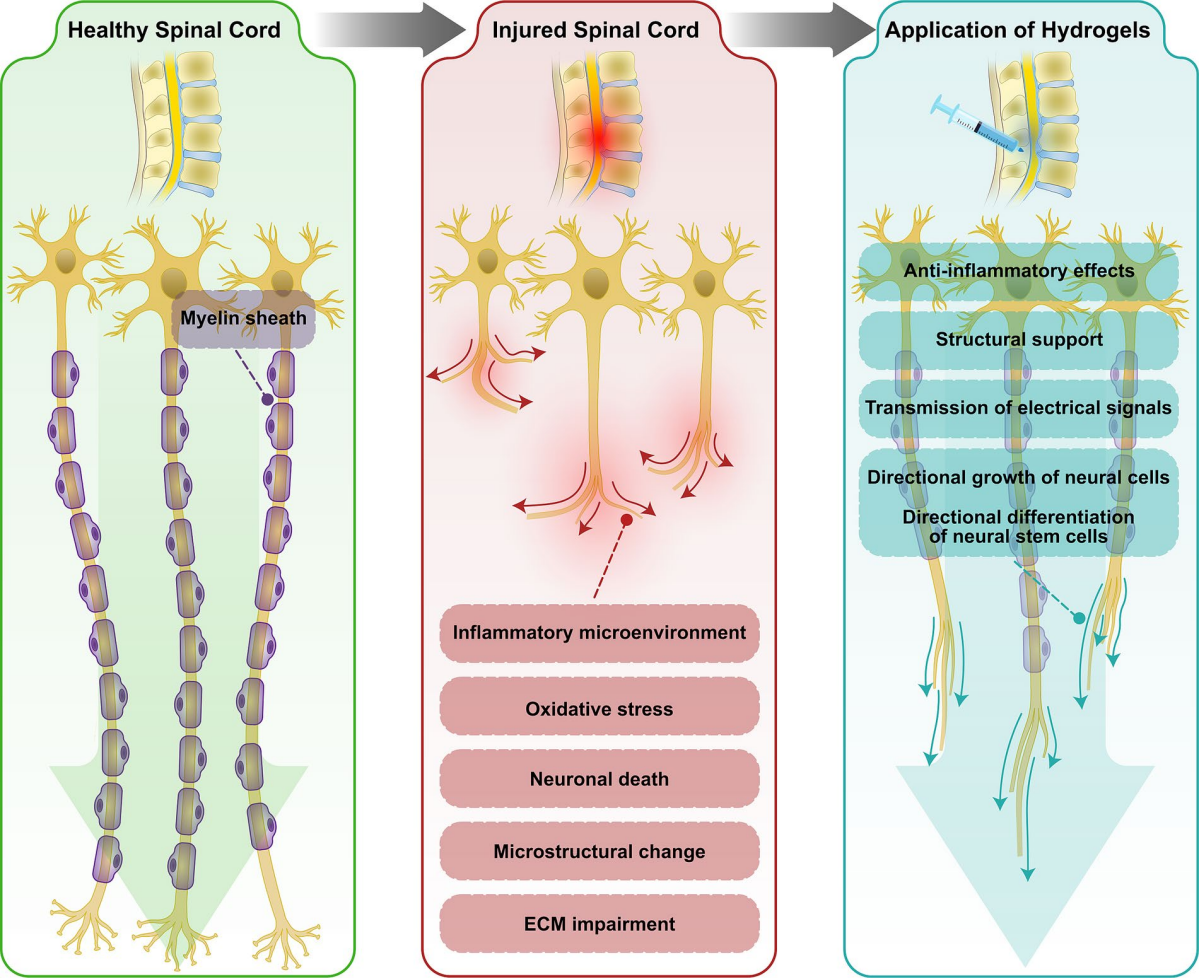
Schematic diagram of spinal cord microstructural disruption after SCI. Spinal cord microstructural disruption after SCI are likely caused by changes in myelin, axonal density, and iron deposition, metabolic alterations, and ECM disruption. Application of hydrogel-encapsulated EVs provides structural support to enhance axonal growth and facilitates the directional growth of neural cells.

In order to overcome structural limitations several biomaterials have been evaluated to be placed into the spinal cord lesion in order to provide a bridge through the spinal cord lesion sites. These biomaterial-based strategies provide structural support to enhance axonal growth and regeneration, promoting motor improvement. This supportive structure facilitates the directional growth of neural cells. In addition, using biomaterials as neuronal bridging would also support cell-based transplants to release neurotrophic factors thus enhancing the therapeutic efficacy of regenerative treatments. Combining biomaterials with cell-based transplants improves biomaterials integration and enhance regenerative growth potential (illustrated in Figure 2; Siebert et al., 2015; Li and Mooney, 2016; Liu et al., 2017).

Spinal ECM is involved in providing structural support and enabling communications under physiological microenvironment. The damaged ECM following SCI contributes to neuroinflammation and impairs neural plasticity. Therefore, spinal cord ECM remodeling could limit the efficacy of regenerative therapies. Biomaterial scaffolds can be used as a solution to counter ECM impairment or loss (Chio et al., 2022). Strategies that use ECM-based biomaterials and combination of cell-derived ECM with synthetic scaffolds have potential to promote tissue repair (Hong et al., 2020; Mazloomnejad et al., 2023). Using combination of biomaterials with cell-based strategies promote nerve injury repair by mimicking the physical and chemical properties of the normal ECM and offering the correct position of neural cell location and ECM deposition (Poongodi et al., 2021).

## 3 Using cell-derived EVs for the regeneration of SCI

EVs are released from many types of cells and contain many biological molecules, such as proteins, lipids, DNA, mRNAs, and miRNAs, which are involved in cell–cell crosstalk. EVs contribute to tissue repair and regeneration and immunosurveillance. Subtypes of EVs are microvesicles, exosomes, and apoptotic bodies, which are classified depending on their biogenesis, release pathways, and size (Samanta et al., 2018; Rezaee et al., 2023). EVs derived from different types of cells promoted functional recovery after SCI through their neuroprotective and neuroregenerative properties (Romanelli et al., 2019). Many studies reported that EVs derived from mesenchymal stem cells (MSCs), neural stem cells (NSCs), astrocytes, macrophages/microglia, and cerebrospinal fluid (CSF) have potential in the treatment of spinal cord injury (Zhong et al., 2020; Dutta et al., 2021; Feng et al., 2021; Zhang et al., 2023). Here we review some possible mechanisms and signaling pathways that might be involved in EVs-induced neuroprotection and neuroregenerative.

The neuroprotective effects of EVs are mainly through inhibition of inflammation, neuronal death, and promotion of neuronal survival (Huang et al., 2017). Macrophages/microglia are one of the main cells participating in neuroinflammation in SCI. Macrophages/microglia polarization toward pro-inflammatory M1 phenotype occurs in response to damaged spinal cord signals (Fu et al., 2022). Human umbilical cord MSCs-EVs (hUCMSCs) attenuate the spinal cord neuroinflammation and regulate the polarization of macrophages/microglia toward the anti-inflammatory M2 phenotype via the A2bR/cAMP/PKA signaling pathway (Zhai et al., 2021). MSCs-EVs also inhibit the TLR4/NF-κB signaling pathway, which promotes M2 macrophage polarization and suppresses inflammation in the spinal cord microenvironment (Jiang and Zhang, 2021; Nie and Jiang, 2021). Similarly, Schwann cell-derived exosomes promote M2 macrophage/microglial polarization and attenuate inflammation by regulating the SOCS3/STAT3 pathway after SCI (Ren et al., 2023). Inflammatory responses may result in apoptosis of neurons and oligodendrocytes and consequent neurological defects (Zhang et al., 2012). NSC-EVs can attenuate apoptosis and inflammatory processes by activating autophagy, which is critical for tissue protection against SCI. They increase the expression of the autophagy proteins and enhance autophagosome formation (Rong et al., 2019a,b). Schwann cell-EVs, MSC-EVs, and microglia-EVs can efficiently assist in neurological recovery after SCI via activation of autophagy and inhibition of apoptosis through inhibition of the Akt/mTOR signaling pathway (Fan et al., 2020; Pan et al., 2022; Gao et al., 2023). Reduced proapoptotic protein Bax, the apoptosis effector cleaved caspase-3, and the pro-inflammatory cytokines TNF-α, IL-1β, and IL-6, and increased anti-apoptotic protein Bcl-2 were reported after EVs transplantation (Rong et al., 2019a; Huang et al., 2020; Chen et al., 2021). In addition, macrophage autophagy is also crucial for M2 polarization, and impaired autophagy results in neuroinflammation (Chen et al., 2017). Peripheral Macrophage-derived exosomes activate autophagy via inhibition of the PI3K/AKT/mTOR pathway, which results in M2-type microglial polarization (Zhang et al., 2021). Studies showed that inflammasome activation is also implicated in neuroinflammation after SCI, and suppressing NLRP3 inflammasome will alleviate neuroinflammation (Jiang et al., 2017). EVs can suppress the formation of NLRP3 inflammasome complex, leading to reduced apoptosis, neural cell regeneration, and improved motor function recovery after SCI (Huang et al., 2020; Mohammed et al., 2020). BSCB integrity is another cause of exacerbated inflammatory reactions and secondary injury (Freyermuth-Trujillo et al., 2022). Lu et al. showed that BMSC-EVs inhibited pericyte migration via suppression of NF-κB p65 signaling pathway leading to decreased BSCB permeability and improved BSCB integrity and consequent reduced neuronal death, increased neuronal survival, and improved motor functions (Lu et al., 2019).

In addition to neuroprotective properties, EVs promote neural regeneration after SCI. EVs derived from hUCMSCs and skin precursor-derived Schwann cells promote axonal growth and neuronal survival by inhibiting PTEN and promoting PI3K/Akt/mTOR pathway (Wu et al., 2020; Xiao et al., 2021). Induced neural progenitor cells-derived EVs also promote the activation of proliferating endogenous neural stem cells by activating the ERK pathway (Ma et al., 2019). Neural repair and tissue regeneration after SCI require normal angiogenesis. EVs have pro-angiogenic properties promoting neurogenesis, axonal remodeling, and improved neurological functions (Zhang et al., 2020; Feng et al., 2021). EVs stimulate angiogenesis by activating PI3K/AKT and Wnt/β-catenin signaling pathway and enhanced VEGF expression after SCI (Rauch et al., 2009; Zhong et al., 2020; Cao et al., 2021; Luo et al., 2021; Huang et al., 2022).

To enhance the therapeutic potential of EVs for targeted spinal cord repair, preconditioning of cell sources and engineering of EVs can be useful (Chen et al., 2022). Hypoxia and melatonin preconditioning of cells alter the contents of EVs toward more protective capacity (Liu et al., 2021; Liang et al., 2022). Hypoxic preconditioning enhances the regulation of some miRNAs that exert neuroprotective properties after SCI through HIF-1a-related pathways (Liu et al., 2020; Huang et al., 2022). Melatonin prevents neural death and facilitates neuroplasticity through attenuating oxidative stress and inflammation after SCI (Xie et al., 2023). Moreover, the specific miRNAs, siRNA, proteins, and small molecular drugs can be loaded into exosomes by engineering strategies to promote targeted repair (Chen et al., 2021; Li et al., 2023; Yaghoobi et al., 2023; Zeng et al., 2023). Therefore, these modified MSC-EVs are promising regenerative agents for SCI.

## 4 Hydrogel-based treatments for SCI regeneration

Hydrogels are a highly porous network of hydrophilic polymers capable of absorbing large amounts of water or other fluids. They are biomaterials commonly utilized in regenerative medicine and appear to be a promising tool especially in the repair of nerve injury, offering a wide range of applications due to their unique properties (Perale et al., 2011). Hydrogels are utilized as scaffolds in regenerative medicine to support the growth and regeneration of cells and tissues. They provide a suitable environment for cells to attach, proliferate, and differentiate, mimicking the natural extracellular matrix (ECM) found in living tissues (Yu et al., 2020). They can be synthesized through crosslinking hydrophilic polymers by physical, chemical, or enzymatic processes, each with its own set of benefits and limitations. The engineering of hydrogels allows for versatility in terms of design and functionality, such as mechanical strength, porosity, biodegradability, and bioactivity. These properties can be tailored to match specific tissue types or applications (Kwon et al., 2012). In the context of CNS injury, hydrogels act as a bridge to connect the two severed ends of the injured nerve, promote axon growth, and provide an attachment site for proliferation and differentiation of transplanted cells. Due to the presence of the BSCB, drug delivery to the CNS might be challenging. Also, the presence of cerebrospinal fluid circulation impedes the colonization of transplanted cells or biological factors, leading to their compromised function. Therefore, designing biomaterials such as hydrogels that are capable of delivering drugs to the damaged site is of great importance. Also, in order to prevent long-term damage to the surrounding tissues, it is crucial for biomaterials designed for CNS repair to degrade within a suitable timeframe (Zhao et al., 2023). Taken together, the use of hydrogels in regenerative medicine, especially in SCI repair, has many advantages, such as the effective delivery of bioactive molecules (such as growth factors or drugs), their biocompatibility with living tissues, and their ability to provide structural support while allowing nutrient exchange (Ji et al., 2014).

Based on their polymer origin, hydrogels can be classified as natural, synthetic, and natural-synthetic composite hydrogels (hybrid hydrogels). Natural hydrogels possess excellent biocompatibility and can mimic the ECM found in tissues, providing a suitable environment for cell growth and tissue regeneration (Perale et al., 2011). Their similarity with the ECM reduces the stimulation of chronic inflammation or immunological reactions and toxicity (Mano et al., 2007). Decellularized ECM (dECM) can be an ideal natural material for the preparation of hydrogels and is expected to play an important role in the future of regeneration therapies (Kasravi et al., 2023). Potential immunogenicity in some natural biomaterials is reported, however there are some considerable practical solutions to overcome the immunogenicity of bioscaffolds (Kasravi et al., 2023). Natural hydrogels can be classified into several types based on their source and materials. Materials are being investigated to support cell therapies are synthesized from polysaccharides such as (alginate, chitosan, and hyaluronic acid), polypeptides such as (collagen), hydrolyzed collagen (such as gelatin) and linked proteins (such as fibrin) (Perale et al., 2011). Synthetic hydrogels, on the other hand, are engineered to have specific properties such as tunable mechanical strength and degradation rates. They can be tailored to mimic the native tissue microenvironment and provide structural support for cells during regeneration. Additionally, hybrid hydrogels combine both natural and synthetic components to harness the advantages of both materials, offering enhanced mechanical properties while maintaining biocompatibility (Cai et al., 2023). These compositions can provide a biocompatible structure for tissue engineering and biomedical applications such as SCI treatment (Riedelová-Reicheltová et al., 2016).

As mentioned previously, hydrogels result from crosslinking polymers through chemical, physical, or enzymatic approaches. While chemical crosslinking is difficult to change, physical crosslinking is reversible, and the state of physical crosslinking might change in response to external conditions (Cai et al., 2023). Crosslinking methods affect the physical properties of the hydrogel, such as stiffness. Hydrogel stiffness exerts important effects on outcomes of different regenerative fields. Soft hydrogels loaded with exosomes showed better nerve repair compared with stiff hydrogels through the quick release of exosomes (Liu et al., 2022). In this regard, smart hydrogels are physically cross-linked hydrogels capable of responding to external stimuli, including changes in temperature, light, electrical signaling, and pH in the environment (Cai et al., 2023). These smart hydrogels have been used in tissue engineering, drug and cell delivery, and the promotion of tissue repair by regulating the tissue microenvironment. Recent studies have reported the beneficial effects of smart hydrogels for SCI treatment. Using smart hydrogels resulted in beneficial effects such as attenuation of inflammatory conditions in SCI (Wang et al., 2021; Zheng et al., 2021). Also, the application of such hydrogels can lead to the release of drugs into the site of SCI, because they can sense and respond to the microenvironment condition (Chang et al., 2022).

Recent studies used modified applications of hydrogels to promote the growth, development, maturation, and regeneration of neural tissue, including the application of conductive hydrogels and incorporation of dopamine or nanomaterials/nanoparticles into hydrogels. Electrical signals play a crucial role in nerve tissue development, growth, and regeneration. Conductive hydrogels can enhance the transmission of electrical signals between adjacent cells, support the reconstruction of impaired signaling pathways, and help to maintain the electrical microenvironment suitable for nerve function and regeneration. Application of these hydrogels in peripheral nerve injury has become a better alternative than the conventional autologous nerve transplantation (Dong et al., 2020). In addition to electrical signals, dopamine plays a significant role in the growth and differentiation of neurons and the promotion of synaptic formation. In this regard, the application of dopamine-functionalized hydrogels can promote neural repair and regeneration. Also, application of such hydrogels promoted the differentiation of NSCs and the growth of synapses (Zhao et al., 2023). Overall, the diverse range of hydrogel materials available in regenerative medicine holds great promise for developing innovative therapies that can effectively repair damaged tissues and organs especially damaged neural tissues.

## 5 Hydrogel-encapsulated exosomes for the regeneration of SCI

EV-based therapies are currently commonly administered through repeated local or intravenous injections. As mentioned earlier, injection of EVs alone may not be appropriate for spinal cord lesions as exosomes clear rapidly and have a short duration of action. Therefore, sustained retention in the lesion site is required to achieve therapeutic effects (Riau et al., 2019). Embedding EVs into hydrogels is applicable for sustained release and effective retention at the site of injury (Chen et al., 2022). A combination of EVs and hydrogels has been assessed as a potential option for the treatment of several neurological conditions. Using hydrogel-integrating EVs in the recovery of motor function in stroke and cerebral ischemia has been suggested (Zhang et al., 2018; Tsintou et al., 2021; Han et al., 2023). It has been shown that the use of hydrogel-loaded exosomes in traumatic brain injury promotes axonal regeneration, remyelination, synapse formation, and remodeling of brain structural and functional recovery through promoting angiogenesis, neurogenesis and microglia immunoregulation (Li et al., 2022; Liu et al., 2023). Exosomes encapsulated in hydrogels can also be used to deliver microRNAs (miRNAs), proteins, and mRNAs to promote functional recovery (Han et al., 2019). In this section, we review preclinical applications of EVs derived from different sources of cells combined with different types of hydrogels and their underlying mechanisms of action (Table 1).

**Table 1:** Preclinical studies of Hydrogel-Encapsulated Extracellular Vesicles for the Regeneration of SCI.

Source of EVs	Type of hydrogel	Characteristic of hydrogel	Delivery route	Animal model	Results	Main involved signaling pathway	Ref.
UCMSC	PLGA-PEG-PLGA	Injectable Temperature Sensitive	Injection into the injury site	Rat	Reduced inflammation Reduced neuronal apoptosis Promoted neurological recovery	Regulating the Nrf2-keap1 signalling cascade Suppressing NLRP3/Caspase-1	Xiao et al. (2023)
CSF	GelMA and hyaluronic acid composite hydrogel	Light responsive	Local application of hydrogel dressing	Mice	Improved motor function recovery Promoted angiogenesis	Activating PI3K/AKT signaling pathway	Li et al. (2023)
NSCs	GelMA	Injectable	Orthotopically injected into the site of injury	Mice	Promoted NSCs differentiation and axonal regeneration Promoted M2 polarization Promoted functional recovery Showed anti-inflammatory effects Promoted axonal regeneration Promoted remyelination	Activating MAPK signaling pathway	Zeng et al. (2023)
MSCs	GelMA hydrogel	Light responsive	Application with MN array patch	Rat	Alleviated the inflammatory response Improved functional recovery	NR	Fang et al. (2023)
MSCs	Hyaluronic acid-based hydrogel	Injectable	Complete spinal cord transection and local implantation of hydrogel	Rat	Improved functional recovery Reduced inflammation Promoted angiogenesis Promoted M2 macrophage polarization	NR	Roh et al. (2023)
hUCMSCs	GelMA	NR	Injection into the injury site	Rat	Reduced neuroinflammation Increased neuroprotection	Activating EGFR/STAT3 signaling	Wang et al. (2023)
hUCMSCs	GelMA	Light responsive Injectable	Injection into the injury site	Rat	Anti-inflammatory effects Anti-fibrotic effect Promoted nerve regeneration Promoted recovery of motor function	NR	Wang et al. (2023)
ADMSCs	Collagen and Fibrin Hydrogel	NR	Local injection into the injury site via Hamilton syringe	Rat	Regenerated the injured nerve Reduced spinal cord lesion-induced central neuropathic pain	NR	Afsartala et al. (2023)
M2 microglia	PLEL hydrogel	Thermosensitive Injectable	Injection into the injury site	Rat	Alleviated inflammation Promote restoration of neuronal function	Inhibiting Bcl-2 pathway	Zhang et al. (2022)
MSC	GelMA hydrogel	NR	Application with MN array patch	Rat	Reduced neuroinflammation Elevated neuroprotective-related proteins and miRNAs	NR	Han et al. (2022)
MSCs	Tannic acid doped hydrogel		Spinal cord transection and local implantation of hydrogel	Rat	Promoted the recovery of motor function Mitigated of the inflammatory and ROS microenvironment	NR	Liu et al. (2022)
BMSCs	GMP hydrogels	Electroconductive	Spinal cord hemisection and local implantation of hydrogel	Mice	Promoted NSC recruitment Promoted axon regeneration Promoted motor recovery	NR	Fan et al. (2022)
BMSCs	Fibrin gel	NR	Transplantation to the lesion site	Mice	Promoted motor function and electrophysiological performance Enhanced neurogenesis Promoted oligodendrogenesis	NR	He et al. (2022)
USC	NR	NR	Local intrathecal injection	Mice	Enhanced spinal cord neurological functional recovery Promoted angiogenesis	Activating PI3K/AKT signaling	Cao et al. (2021)
hUCMSCs	Hyaluronic acid hydrogel	Adhesive	Transplantation to the lesion site	Rat	Promotes angiogenesis Nerve regeneration at The site of spinal cord injury Locomotor function recovery	Activating HIF-1α/VEGF	Mu et al. (2022)
hUC-MSCs	Fibrin glue	NR	Spinal cord transection and local implantation of hydrogel	Rat	Alleviated the inflammatory and oxidative microenvironment Induced effective nerve tissue repair and functional recovery	NR	Mu et al. (2021)
ADMSCs	F127-polycitrate-polyethyleneimine hydrogel (FE)	Thermosensitive Injectable Adhesive	Spinal cord transection and local implantation of hydrogel	Rat	Reduced fibrotic scar formation Reduced inflammatory reaction Promoted remyelination and axonal generation Reduced neurons apoptosis Enhanced motor function Suppressed the cavity formation after SCI	NR	Wang et al. (2021)
BMSCs	3D gelatin methacrylate hydrogel (GelMA)	NR	Spinal cord transection and local implantation of hydrogel	Rat	Promoted neuronal differentiation and extension Facilitated nerve regeneration Reduced glial scarring	NR	Cheng et al. (2021)
M2 macrophage	NR	Photocurable	Local implantation of hydrogel	Mice	Modulated vascular regeneration Promoted neurological functional recovery	Activating Wnt/β-catenin pathway	Luo et al. (2021)
hpAMSCs	Peptide-modified hydrogel (PGel)	Adhesive	Spinal cord transection and local implantation of hydrogel	Rat	Promoted antioxidant and anti-inflammatory effects on the microenvironment Promoted nerve repair	NR	Li et al. (2020)

MSCs, mesenchymal stem cells; UCMSC, umbilical cord mesenchymal stem cells; CSF, cerebrospinal fluid; NSCs, neural stem cells; ADMSCs, adipose-mesenchymal derived stem cells; BMSCs, bone marrow-derived mesenchymal stem cells; USC, urine-derived stem cells; hpAMSCs, human placenta amnion derived mesenchymal stem cells; NR, not reported.

The most common source of EVs was MSCs, which were used in combination with different types of hydrogels to repair injured nerve tissue. Many studies reported that using EVs derived from different types of MSCs can inhibit neuroinflammation and prevent the process of secondary injury after SCI (Sun et al., 2018; Wang et al., 2018; Lu et al., 2019). In a recent study, Roh et al. used a hyaluronic acid-based hydrogel combined with biochemical cues containing MSCs-derived EVs for neuronal regeneration after SCI. This composite scaffold enhanced spinal cord regeneration by promoting angiogenesis, neural differentiation, and remyelination, inhibiting inflammation and apoptosis and also reducing glial scarring. It induced M2 macrophage polarization and decreased M1 macrophage-related inflammation, resulting in enhanced functional recovery (Roh et al., 2023).

BMSC and their EVs had beneficial neuroprotective effects on tissue repair and axonal growth. Application of BMSC and their EVs resulted in attenuated neuronal apoptosis and inhibited inflammatory response during SCI through inhibiting the TLR4/MyD88/NF-κB signaling pathway, caspase-3, and 12 (Ide et al., 2010; Novikova et al., 2011; Bai et al., 2019; Gu et al., 2020; Fan et al., 2021). In a study by Fan et al. electroconductive hydrogels encapsulated BMSCs-exosomes enhanced tissue repair and significant functional recovery after SCI. These exosome-loaded hydrogels activate the PTEN/PI3K/AKT/mTOR pathway, leading to myelin-associated axonal regeneration, enhanced neurogenesis, and reduced neuroinflammation. On the other hand, GMP hydrogel increased the proportion of M1 microglia through activation of the IKKα/β/ NF-κB pathway in the inflammatory microenvironment of the damaged spinal cord, which causes aggravation of neuroinflammation. Combining BMSCs-exosomes with GMP hydrogel promoted polarization of microglia into M2 phenotype via decreasing expression of phosphorylated-IKKα/β, p-IκBα, and p-P65 (Fan et al., 2022). He et al. showed that BMSC-derived exosomes loaded in fibrin gel enhanced oligodendrogenesis, as expression of neuronal markers such as doublecortin (Dcx), NeuN, and Tuj1 were significantly increased. These exosomes contained high levels of neuropeptide VGF. VGF promotes myelination and oligodendrogenesis, resulting in functional recovery (He et al., 2022). Neurological functional recovery after BMSC-derived exosomes encapsulated in 3D gelatin methacrylate hydrogel (GelMA) has also been reported in another study. GelMA-Exos promoted neuronal differentiation and reduced glial scars in injured sites (Cheng et al., 2021).

Transplantation of hUCMSCs and their EVs can promote SCI repair by attenuating inflammatory responses and promoting nerve regeneration. These neuroprotective properties of hUCMSCs were related to the activation of the PTEN/Akt/mTOR axis, decreased inflammatory cytokines, increased anti-inflammatory cytokines, and activation of M2 macrophages (Bao et al., 2018; Xiao et al., 2021; Zhu et al., 2021; Sun et al., 2023). hUCMSCs derived exosomes encapsulated in fibrin glue resulted in nerve tissue regeneration as they reduced lesion volume, increased integrity, improved remyelination, a more abundant distribution of neurofilament proteins (indicators of mature neurons) in both the lesion and adjacent areas, and also significantly increased distribution of choline acetyltransferase (an indicator for cholinergic neurons). These fibrin glue exosomes also inhibited excessive production of reactive oxygen species (ROS) and prevented the oxidative procedures. They exhibited significant anti-inflammatory effects via the regulation of macrophage phenotypes toward M2 macrophages (Mu et al., 2021). In addition, four-dimensional cultured hUCMSC-derived sEVs combined with GelMA hydrogel attenuated neuroinflammation through polarization of the macrophage/microglia from M1 to M2 phenotype, resulting in enhanced survival of spinal neurons after injury. These EVs promoted polarization of the M2 phenotype via upregulation of IGFBP2/EGFR protein, leading to phosphorylation of STAT3 (Wang et al., 2023). Another study used a combination of Berberine-loaded hUC-MSCs-sEVs with GelMA hydrogel, which resulted in attenuated local inflammation and created a favorable microenvironment for nerve regeneration and axonal regrowth (Wang et al., 2023). A tannic acid-doped hydrogel with sustainable hUCMSCs-derived sEV release has also been shown to exhibit anti-inflammatory and anti-antioxidant properties leading to restoration of motor function and urinary tissue preservation in SCI (Liu et al., 2022). As mentioned earlier, exosomes can be loaded with different substances to promote SCI recovery. miR-138-5p-loaded UCMSC-exosomes combined with a temperature-sensitive hydrogel showed promising results. Protective effects of miR-138-5p-loaded exosomes were reported in an *in vitro* assessment. These exosomes were able to reduce neuronal apoptosis through the reduction of intracellular ROS production and consequent oxidative stress via activation of the Nrf2-mediated pathway. They also reduced inflammation through suppression of the NLRP3-caspase1 signaling pathway and inhibition of M1 polarization. In addition, similarly *in vivo* experiments showed that Exos-138 hydrogel can promote axonal regeneration, and motor function recovery and reduce inflammation in rats with SCI (Xiao et al., 2023). Hypoxia preconditioned hUCMSCs-derived exosomes loaded by adhesive hyaluronic acid hydrogel promoted angiogenesis, nerve regeneration, and functional recovery after SCI. These regenerative effects were achieved by activation of HIF-1α and overexpression of VEGF (Mu et al., 2022). Hypoxic preconditioning of mesenchymal stem cells enhances neuroprotection and ameliorates SCI via improved survival and migration of cells, angiogenesis, and decreased neuroinflammation via HIF-1α related signaling pathways (Imura et al., 2017; Wang et al., 2018). In addition, hypoxia-preconditioned MSC-derived EVs were reported to have higher regenerative capacity than those obtained under normoxia. Priming MSC in hypoxia favors generating EVs enriched in HIF-1α and hypoxia regulated-miRNAs (HRMs), leading to attenuated inflammatory/oxidative stress (Luo et al., 2019; Huang et al., 2022; Pulido-Escribano et al., 2022). Similarly, exosomes derived from hypoxic preconditioned human umbilical vein endothelial cells (HUVEC) stimulated MSCs for angiogenic SCI treatment. Application of these MSCs in a hyaluronic acid hydrogel scaffold resulted in an effective nerve tissue repair and significant motor function recovery (Li et al., 2022).

Adipose tissue-derived mesenchymal stem cells (ADMSCs) are another type of MSCs that was used as a source of exosomes. ADMSCs and ADMSCs-derived EVs can improve neurological function by reducing inflammatory responses and regulating microglial polarization (Mukhamedshina et al., 2018; Chen et al., 2022; Sung et al., 2022; Luo et al., 2023). The combination of ADMSCs-derived EVs with F127-polycitrate-polyethyleneimine hydrogel (FE) promoted motor functional recovery of spinal cord injury. This type of hydrogel was reported to be multifunctional as they were injectable, adhesive, temperature-responsive, self-healing, and anti-inflammatory. Using FE hydrogel to control the sustained release of EVs at the spinal cord injured site promoted functional recovery through attenuating inflammatory reaction, promoting remyelination and axonal regeneration, and reduced neuron apoptosis after SCI. FE-EVs also reduced the fibrotic scar formation via suppressing fibroblast migration, leading to better axonal regeneration in the microenvironment. They can also reduce excessive activated macrophages, resulting in enhanced remyelination and axonal regeneration (Wang et al., 2021). Transplantation of ADMSCs-EVs encapsulated within collagen and fibrin hydrogel (ECM-based hydrogel scaffolds) can regenerate the injured nerve and decrease SCI-induced central neuropathic pain (Afsartala et al., 2023).

Human placenta amniotic membrane mesenchymal stem cells (hpAMSCs) isolated from the mesenchymal layer of the human placenta amniotic membrane are another potential therapeutic option in the repair of spinal cord injury. hpAMSCs and their EVs exert neuroprotective effects after SCI through reactivation of endogenous neurogenesis, axonal regeneration, promotion of angiogenesis, and reductions of inflammatory cell infiltration and cell apoptosis (Zhou et al., 2016, 2021; Zhang et al., 2020). hpAMSCs-derived exosomes loaded in a peptide-modified adhesive hydrogel (Exo-pGel) promoted significant nerve recovery and urinary tissue preservation by effectively reducing inflammation and oxidation. Significant reduction in inducible Nitric Oxide Synthase, (iNOS), peroxidative agents, and neuronal death were reported in the Exo-pGel treatment. Higher densities of neurofilaments and choline acetyltransferase and lesser accumulation of astrocytes in the Exo-pGel treatment indicated regeneration of nerves and restricted secondary damage (Li et al., 2020).

Instead of direct injection of MSCs or MSC-EVs into the injured site, Fang et al. fabricated microneedle (MN) array-MSC patches seeded with MSCs to achieve better and longer sustained delivery of MSC-EVs. Localized and sustained release of MSC-EVs after MN patch implantation was reported at the spinal injury site. The solution of MSC-EVs mixed with GelMA was mounted on the top of the MN patch, followed by a blue light photocuring process. MN-MSC seeded patches effectively downregulated neuroinflammation via reducing inflammatory factors (TNF-a, IL-1ß, MMP-9) and inflammatory immune cells (M1 microglia), increasing anti-inflammatory factors (TGF-ß, Arg-2). More neurons, less apoptosis of spinal cord cells, more myelin sheath, more axon regrowth, and more protection of spared axons from secondary injury were reported in the treatment with MN-MSC patch, resulting in functional recovery (Fang et al., 2023). The hybrid of GelMA hydrogel-MN array patch with 3D-cultured MSC-derived exosomes was used in another study for spinal cord repair. This hybrid reduced the number of apoptotic cells, improved spinal cord tissue morphology (significant reduction of cavity volume), and effectively attenuated neuroinflammation via promoting polarization of microglia from the M1 to M2 phenotype in the injured site (Han et al., 2022). As both studies mentioned, the advantage of using these microneedle (MN) array patches was the sustained release of EVs into the injured microenvironment (Han et al., 2022; Fang et al., 2023).

As mentioned earlier, preconditioning may have beneficial effects on EV production and quality. Zeng et al. reported neuroprotective and neurodegenerative properties of EVs derived from melatonin-pretreated NSCs (Zeng et al., 2023). Melatonin preconditioning promotes the anti-inflammatory, antioxidant, and antiapoptotic properties of stem cells derived-EVs. These effects are mainly due to increased polarization of microglia/macrophages from M1 to M2, reduced the ROS production and regulated mitochondrial function (Liu et al., 2021). Transplantation of these EVs combined with GelMA exerted anti-inflammatory effects and promoted NSCs differentiation, axonal regeneration, M2 polarization, remyelination, and functional recovery (Zeng et al., 2023). Different conditioning methods need to be further investigated in EVs-hydrogel combination therapies to improve the quality of EVs.

Local administration of human urine stem cell (USC)-derived exosomes enriched with ANGPTL3 embedded in hydrogel can improve neurological functional recovery after spinal cord injury through enhancing angiogenesis. After crossing the blood–brain barrier (BBB), USC-Exos is taken up by endothelial cells in the injured site, and ANGPTL3 acts as an angiogenic mediator via activation of the PI3K/AKT signaling pathway in these cells. Obtaining USC-Exos is a non-invasive approach and easily assessable, which can be a new innovative therapeutic strategy (Cao et al., 2021).

Another source that was used in studies was CSF-EVs derived from the subarachnoid space of SCI pigs. SCI-CSF-EVs are suggested to be more associated with neurological functional recovery following SCI in comparison with EVs derived from other sources. These EVs contain protective factors secreted by spinal cord cells, which are involved in the promotion of the neurological function after SCI. Li et al. shown that using these EVs promotes vascular regeneration and recovery of motor and sensory function mainly through activating of the PI3K/AKT pathway (Li et al., 2023). PI3K/AKT is an important signaling pathway for neurological functional recovery after spinal cord injury. This pathway is crucial for neuron repair, angiogenesis promotion, and microenvironment improvement. This pathway is also involved in promoting macrophage polarization toward M2 phenotype and microglial proliferation (Xu et al., 2021; Wang et al., 2022; Li et al., 2023).

M2 macrophages are another potential source of exosomes that can enhance angiogenesis, neurogenesis, and tissue regeneration after SCI (Huang et al., 2022). Hydrogel-mediated sustained release of M2-Exos significantly promoted vascular regeneration through activating Wnt/β-catenin signaling and inducing the expression of angiogenic-related genes in spinal cord microvascular endothelial cells, resulting in functional neurological recovery after SCI (Luo et al., 2021). Activation of the HIF-1α/VEGF axis is also involved in M2-Exos-induced angiogenesis and functional recovery following SCI (Huang et al., 2022). Zhang et al. have also demonstrated the functional role of M2 microglia-derived EVs in SCI therapy. The incorporation of M2-EVs into PLEL hydrogel resulted in improved motor nerve functional recovery through increased BBB scores and reduced scare areas. They were involved in the reduction of local inflammation through EVs-induced M2 polarization and reduced neuronal apoptosis through inhibition of the Bcl-2 pathway (Zhang et al., 2022).

## 6 Conclusion and perspectives

To date, there are no fully effective treatment methods for regeneration of the spinal cord after SCI due to the complex microenvironment of the injured spinal cord. There are currently some clinical trials assessing the safety and efficacy of EVs in patients with CNS conditions (e.g., ischemic stroke, depression, anxiety, and dementias) and also some clinical trials using hydrogel-based strategies in the treatment of SCI. The novel EVs-hydrogels combination therapy can be a promising therapeutic strategy to promote neuronal survival, axonal regeneration, and tissue remodeling by delivering EVs directly to the injured site and providing a supportive scaffold for tissue repair. A combination of tissue engineering strategies with rehabilitation and neuromodulation approaches may help to enhance the efficacy of spinal cord regeneration. This approach can be used as a personalized strategy by selecting the appropriate cell source and engineering the optimum exosomes. However, there are still some issues that are necessary to be addressed before clinical application, so further preclinical studies are needed. These challenges include ensuring the long-term and sustained safety and efficacy of EVs and optimizing the hydrogel properties. The quality of EVs can be improved through different methods such as biophysical inducing, biochemical preconditioning, and cellular reprogramming of EV cell sources. Genetic reprogramming of cells, such as overexpression of specific proteins and miRNA in cells, can increase the therapeutic potency of secreted EVs. In addition, decreasing the stiffness of hydrogel, reducing the complexity of hydrogel preparation, and providing user-friendly hydrogels with controllable and predictable gelation time should also be considered.

Taken together, this strategy provides a homeostatic environment that would mimic the physiologic niche of the spinal cord. Indeed, therapeutic strategies of hydrogel-loaded EVs still have a long way to go, from pre-clinical experiments to clinical application. The focus of future preclinical studies and clinical trials should be on assessing the long-term safety and efficacy of the best cell source and hydrogel type to provide complete spinal cord regeneration.

## Author contributions

YN: Conceptualization, Investigation, Writing – original draft, Writing – review & editing. AN: Conceptualization, Investigation, Writing – original draft, Writing – review & editing. FM-J: Conceptualization, Investigation, Writing – original draft, Writing – review & editing. PS: Conceptualization, Investigation, Writing – original draft, Writing – review & editing. MJ: Conceptualization, Investigation, Supervision, Writing – original draft, Writing – review & editing. SJ: Conceptualization, Investigation, Supervision, Writing – original draft, Writing – review & editing.

## References

[ref1] Afsartala Z. Hadjighassem M. Shirian S. Ebrahimi-Barough S. Gholami L. Parasmanesh G.. (2023). The effect of collagen and fibrin hydrogels encapsulated with adipose tissue mesenchymal stem cell-derived exosomes for treatment of spinal cord injury in a rat model. Iran. J. Biotechnol. 21, 1–13. doi: 10.30498/IJB.2023.362229.3505 PMC10858360 38344702

[ref2] Ahuja C. S. Wilson J. R. Nori S. Kotter M. R. N. Druschel C. Curt A.. (2017). Traumatic spinal cord injury. Nat. Rev. Dis. Primers. 3:17018. doi: 10.1038/nrdp.2017.18 28447605

[ref3] Amirzadeh gougheri K. Ahmadi A. Ahmadabadi M. G. Babajani A. Yazdanpanah G. Bahrami S.. (2023). Exosomal cargo: pro-angiogeneic, anti-inflammatory, and regenerative effects in ischemic and non-ischemic heart diseases – a comprehensive review. Biomed. Pharmacother. 168:115801. doi: 10.1016/j.biopha.2023.115801, PMID: 37918257

[ref4] Anjum A. Yazid M. D.’. Fauzi Daud M. Idris J. Ng A. M. H. Selvi Naicker A.. (2020). Spinal cord injury: pathophysiology, multimolecular interactions, and underlying recovery mechanisms. Int. J. Mol. Sci. 21:7533. doi: 10.3390/ijms21207533, PMID: 33066029 PMC7589539

[ref5] Ashammakhi N. Kim H. J. Ehsanipour A. Bierman R. D. Kaarela O. Xue C.. (2019). Regenerative therapies for spinal cord injury. Tissue Eng. Part B Rev. 25, 471–491. doi: 10.1089/ten.teb.2019.0182, PMID: 31452463 PMC6919264

[ref6] Bai S. Zhou H. Wu L. (2019). Bone marrow stromal cells improved functional recovery in spinal cord injury rats partly via the toll-like receptor-4/nuclear factor-κB signaling pathway. Exp. Ther. Med. 17, 444–448. doi: 10.3892/etm.2018.6907, PMID: 30651819 PMC6307380

[ref7] Bao C. S. Li X. L. Liu L. Wang B. Yang F. B. Chen L. G. (2018). Transplantation of human umbilical cord mesenchymal stem cells promotes functional recovery after spinal cord injury by blocking the expression of IL-7. Eur. Rev. Med. Pharmacol. Sci. 22, 6436–6447. doi: 10.26355/eurrev_201810_16056, PMID: 30338812

[ref8] Burns T. C. Quinones-Hinojosa A. (2021). Regenerative medicine for neurological diseases-will regenerative neurosurgery deliver? BMJ 373:n955. doi: 10.1136/bmj.n955 34162530

[ref9] Cai M. Chen L. Wang T. Liang Y. Zhao J. Zhang X.. (2023). Hydrogel scaffolds in the treatment of spinal cord injury: a review. Front. Neurosci. 17:1211066. doi: 10.3389/fnins.2023.1211066, PMID: 37325033 PMC10266534

[ref10] Cao Y. Xu Y. Chen C. Xie H. Lu H. Hu J. (2021). Local delivery of USC-derived exosomes harboring ANGPTL3 enhances spinal cord functional recovery after injury by promoting angiogenesis. Stem Cell Res Ther 12, 1–17. doi: 10.1186/s13287-020-02078-8 33413639 PMC7791988

[ref11] Chang S. Wang S. Liu Z. Wang X. (2022). Advances of stimulus-responsive hydrogels for bone defects repair in tissue engineering. Gels 8:389. doi: 10.3390/gels8060389, PMID: 35735733 PMC9222548

[ref12] Chen Z. Bachhuka A. Han S. Wei F. Lu S. Visalakshan R. M.. (2017). Tuning chemistry and topography of Nanoengineered surfaces to manipulate immune response for bone regeneration applications. ACS Nano 11, 4494–4506. doi: 10.1021/acsnano.6b07808, PMID: 28414902

[ref13] Chen Y. Lin J. Yan W. (2022). A prosperous application of hydrogels with extracellular vesicles release for traumatic brain injury. Front. Neurol. 13:908468. doi: 10.3389/fneur.2022.908468, PMID: 35720072 PMC9201053

[ref14] Chen S. Sun F. Qian H. Xu W. Jiang J. (2022). Preconditioning and engineering strategies for improving the efficacy of mesenchymal stem cell-derived exosomes in cell-free therapy. Stem Cells Int. 2022:1779346. doi: 10.1155/2022/1779346 35607400 PMC9124131

[ref15] Chen Y. Tian Z. He L. Liu C. Wang N. Rong L.. (2021). Exosomes derived from miR-26a-modified MSCs promote axonal regeneration via the PTEN/AKT/mTOR pathway following spinal cord injury. Stem Cell Res Ther 12:224. doi: 10.1186/s13287-021-02282-0, PMID: 33820561 PMC8022427

[ref16] Chen J. Wang L. Liu M. Gao G. Zhao W. Fu Q.. (2022). Implantation of adipose-derived mesenchymal stem cell sheets promotes axonal regeneration and restores bladder function after spinal cord injury. Stem Cell Res Ther 13:503. doi: 10.1186/s13287-022-03188-1, PMID: 36224621 PMC9558366

[ref17] Chen J. Zhang C. Li S. Li Z. Lai Z. Xia Q. (2021). Exosomes derived from nerve stem cells loaded with FTY720 promote the recovery after spinal cord injury in rats by PTEN/AKT signal pathway. J Immunol Res 2021:8100298. doi: 10.1155/2021/8100298 34337080 PMC8294984

[ref18] Cheng J. Chen Z. Liu C. Zhong M. Wang S. Sun Y.. (2021). Bone mesenchymal stem cell-derived exosome-loaded injectable hydrogel for minimally invasive treatment of spinal cord injury. Nanomedicine (Lond.) 16, 1567–1579. doi: 10.2217/nnm-2021-0025, PMID: 34189939

[ref19] Chio J. C. T. Punjani N. Hejrati N. Zavvarian M. M. Hong J. Fehlings M. G. (2022). Extracellular matrix and oxidative stress following traumatic spinal cord injury: physiological and pathophysiological roles and opportunities for therapeutic intervention. Antioxid. Redox Signal. 37, 184–207. doi: 10.1089/ars.2021.0120, PMID: 34465134

[ref20] Dong M. Shi B. Liu D. Liu J. H. Zhao D. Yu Z. H.. (2020). Conductive hydrogel for a photothermal-responsive stretchable artificial nerve and coalescing with a damaged peripheral nerve. ACS Nano 14, 16565–16575. doi: 10.1021/acsnano.0c05197, PMID: 33025785

[ref21] Donovan J. Kirshblum S. (2018). Clinical trials in traumatic spinal cord injury. Neurotherapeutics 15, 654–668. doi: 10.1007/s13311-018-0632-5 29736858 PMC6095794

[ref22] Dutta D. Khan N. Wu J. Jay S. M. (2021). Extracellular vesicles as an emerging frontier in spinal cord injury pathobiology and therapy. Trends Neurosci. 44, 492–506. doi: 10.1016/j.tins.2021.01.003, PMID: 33581883 PMC8159852

[ref23] Fan L. Dong J. He X. Zhang C. Zhang T. (2021). Bone marrow mesenchymal stem cells-derived exosomes reduce apoptosis and inflammatory response during spinal cord injury by inhibiting the TLR4/MyD88/NF-κB signaling pathway. Hum. Exp. Toxicol. 40, 1612–1623. doi: 10.1177/09603271211003311, PMID: 33779331

[ref24] Fan Y. Li Y. Huang S. Xu H. Li H. Liu B. (2020). Resveratrol-primed exosomes strongly promote the recovery of motor function in SCI rats by activating autophagy and inhibiting apoptosis via the PI3K signaling pathway. Neurosci. Lett. 736:135262. doi: 10.1016/j.neulet.2020.135262, PMID: 32682847

[ref25] Fan L. Liu C. Chen X. Zheng L. Zou Y. Wen H.. (2022). Exosomes-loaded Electroconductive hydrogel synergistically promotes tissue repair after spinal cord injury via Immunoregulation and enhancement of myelinated axon growth. Adv Sci (Weinh) 9:e2105586. doi: 10.1002/advs.202105586, PMID: 35253394 PMC9069372

[ref26] Fang A. Wang Y. Guan N. Zuo Y. Lin L. Guo B.. (2023). Porous microneedle patch with sustained delivery of extracellular vesicles mitigates severe spinal cord injury. Nat. Commun. 14:4011. doi: 10.1038/s41467-023-39745-2, PMID: 37419902 PMC10328956

[ref27] Feng J. Zhang Y. Zhu Z. Gu C. Waqas A. Chen L. (2021). Emerging exosomes and Exosomal MiRNAs in spinal cord injury. Front. Cell Dev. Biol. 9:9. doi: 10.3389/fcell.2021.703989 PMC8299525 34307384

[ref28] Flack J. A. Sharma K. D. Xie J. Y. (2022). Delving into the recent advancements of spinal cord injury treatment: a review of recent progress. Neural Regen. Res. 17, 283–291. doi: 10.4103/1673-5374.317961, PMID: 34269189 PMC8463999

[ref29] Freyermuth-Trujillo X. Segura-Uribe J. J. Salgado-Ceballos H. Orozco-Barrios C. E. Coyoy-Salgado A. (2022). Inflammation: a target for treatment in spinal cord injury. Cells 11:2692. doi: 10.3390/cells11172692, PMID: 36078099 PMC9454769

[ref30] Fu S. P. Chen S. Y. Pang Q. M. Zhang M. Wu X. C. Wan X.. (2022). Advances in the research of the role of macrophage/microglia polarization-mediated inflammatory response in spinal cord injury. Front. Immunol. 13:1014013. doi: 10.3389/fimmu.2022.1014013, PMID: 36532022 PMC9751019

[ref31] Ganau M. (2014). Tackling gliomas with nanoformulated antineoplastic drugs: suitability of hyaluronic acid nanoparticles. Clin. Transl. Oncol. 16, 220–223. doi: 10.1007/s12094-013-1114-1 24072561

[ref32] Ganau M. Syrmos N. C. D'Arco F. Ganau L. Chibbaro S. Prisco L.. (2017). Enhancing contrast agents and radiotracers performance through hyaluronic acid-coating in neuroradiology and nuclear medicine. Hell. J. Nucl. Med. 20, 166–168. doi: 10.1967/s002449910558, PMID: 28697194

[ref33] Gao X. Gao L. F. Kong X. Q. Zhang Y. N. Jia S. Meng C. Y. (2023). Mesenchymal stem cell-derived extracellular vesicles carrying miR-99b-3p restrain microglial activation and neuropathic pain by stimulating autophagy. Int. Immunopharmacol. 115:109695. doi: 10.1016/j.intimp.2023.109695, PMID: 36638658

[ref34] Ghasempour E. Hesami S. Movahed E. keshel S. H. Doroudian M. (2022). Mesenchymal stem cell-derived exosomes as a new therapeutic strategy in the brain tumors. Stem Cell Res Ther 13:527. doi: 10.1186/s13287-022-03212-4, PMID: 36536420 PMC9764546

[ref35] Gu J. Jin Z. S. Wang C. M. Yan X. F. Mao Y. Q. Chen S. (2020). Bone marrow mesenchymal stem cell-derived exosomes improves spinal cord function after injury in rats by activating autophagy. Drug Des. Devel. Ther. 14, 1621–1631. doi: 10.2147/DDDT.S237502, PMID: 32425507 PMC7196809

[ref36] Guo Y. Gao F. Liu Y. Guo H. Yu W. Chen Z.. (2019). White matter microstructure alterations in patients with spinal cord injury assessed by diffusion tensor imaging. Front. Hum. Neurosci. 13:11. doi: 10.3389/fnhum.2019.00011, PMID: 30809136 PMC6379286

[ref37] Hall A. Fortino T. Spruance V. Niceforo A. Harrop J. S. Phelps P. E.. (2022). Cell transplantation to repair the injured spinal cord. Int. Rev. Neurobiol. 166, 79–158. doi: 10.1016/bs.irn.2022.09.008, PMID: 36424097 PMC10008620

[ref38] Han M. Yang H. Lu X. Li Y. Liu Z. Li F.. (2022). Three-dimensional-cultured MSC-derived exosome-hydrogel hybrid microneedle Array patch for spinal cord repair. Nano Lett. 22, 6391–6401. doi: 10.1021/acs.nanolett.2c02259, PMID: 35876503

[ref39] Han M. Zhang Z. Liu Z. Liu Y. Zhao H. Wang B.. (2023). Three-dimensional-cultured MSC-derived exosome with hydrogel for cerebral ischemia repair. Biomater Adv 149:213396. doi: 10.1016/j.bioadv.2023.213396, PMID: 37011424

[ref40] Han C. Zhou J. Liu B. Liang C. Pan X. Zhang Y.. (2019). Delivery of miR-675 by stem cell-derived exosomes encapsulated in silk fibroin hydrogel prevents aging-induced vascular dysfunction in mouse hindlimb. Mater. Sci. Eng. C Mater. Biol. Appl. 99, 322–332. doi: 10.1016/j.msec.2019.01.122, PMID: 30889706

[ref41] He X. Yang L. Dong K. Zhang F. Liu Y. Ma B.. (2022). Biocompatible exosome-modified fibrin gel accelerates the recovery of spinal cord injury by VGF-mediated oligodendrogenesis. J. Nanobiotechnol. 20:360. doi: 10.1186/s12951-022-01541-3, PMID: 35918769 PMC9344707

[ref42] Hejrati N. Fehlings M. G. (2021). A review of emerging neuroprotective and neuroregenerative therapies in traumatic spinal cord injury. Curr. Opin. Pharmacol. 60, 331–340. doi: 10.1016/j.coph.2021.08.009, PMID: 34520943

[ref43] Hejrati N. Wong R. Khazaei M. Fehlings M. G. (2023). How can clinical safety and efficacy concerns in stem cell therapy for spinal cord injury be overcome? Expert. Opin. Biol. Ther. 23, 883–899. doi: 10.1080/14712598.2023.2245321, PMID: 37545020

[ref44] Hellenbrand D. J. Quinn C. M. Piper Z. J. Morehouse C. N. Fixel J. A. Hanna A. S. (2021). Inflammation after spinal cord injury: a review of the critical timeline of signaling cues and cellular infiltration. J. Neuroinflammation 18:284. doi: 10.1186/s12974-021-02337-2, PMID: 34876174 PMC8653609

[ref45] Hong J. Y. Seo Y. Davaa G. Kim H. W. Kim S. H. Hyun J. K. (2020). Decellularized brain matrix enhances macrophage polarization and functional improvements in rat spinal cord injury. Acta Biomater. 101, 357–371. doi: 10.1016/j.actbio.2019.11.012, PMID: 31711898

[ref46] Huang J.-H. Fu C. H. Xu Y. Yin X. M. Cao Y. Lin F. Y. (2020). Extracellular vesicles derived from epidural fat-mesenchymal stem cells attenuate NLRP3 Inflammasome activation and improve functional recovery after spinal cord injury. Neurochem. Res. 45, 760–771. doi: 10.1007/s11064-019-02950-x, PMID: 31953741

[ref47] Huang J. H. He H. Chen Y. N. Liu Z. Romani M. D. Xu Z. Y.. (2022). Exosomes derived from M2 macrophages improve angiogenesis and functional recovery after spinal cord injury through HIF-1α/VEGF Axis. Brain Sci. 12:1322. doi: 10.3390/brainsci12101322, PMID: 36291255 PMC9599527

[ref48] Huang T. Jia Z. Fang L. Cheng Z. Qian J. Xiong F.. (2022). Extracellular vesicle-derived miR-511-3p from hypoxia preconditioned adipose mesenchymal stem cells ameliorates spinal cord injury through the TRAF6/S1P axis. Brain Res. Bull. 180, 73–85. doi: 10.1016/j.brainresbull.2021.12.015, PMID: 34974133

[ref49] Huang J. H. Yin X. M. Xu Y. Xu C. C. Lin X. Ye F. B.. (2017). Systemic Administration of Exosomes Released from mesenchymal stromal cells attenuates apoptosis, inflammation, and promotes angiogenesis after spinal cord injury in rats. J. Neurotrauma 34, 3388–3396. doi: 10.1089/neu.2017.5063, PMID: 28665182

[ref50] Ide C. Nakai Y. Nakano N. Seo T. B. Yamada Y. Endo K.. (2010). Bone marrow stromal cell transplantation for treatment of sub-acute spinal cord injury in the rat. Brain Res. 1332, 32–47. doi: 10.1016/j.brainres.2010.03.043 20307513

[ref51] Imai T. Takahashi Y. Nishikawa M. Kato K. Morishita M. Yamashita T.. (2015). Macrophage-dependent clearance of systemically administered B16BL6-derived exosomes from the blood circulation in mice. J Extracell Vesicles 4:26238. doi: 10.3402/jev.v4.26238, PMID: 25669322 PMC4323410

[ref52] Imura T. Tomiyasu M. Otsuru N. Nakagawa K. Otsuka T. (2017). Hypoxic preconditioning increases the neuroprotective effects of mesenchymal stem cells in a rat model of spinal cord injury. Journal of Stem Cell Research & Therapy 7, 1–7. doi: 10.4172/2157-7633.1000375

[ref53] Ji W. Hu S. Zhou J. Wang G. Wang K. Zhang Y. (2014). Tissue engineering is a promising method for the repair of spinal cord injuries (review). Exp. Ther. Med. 7, 523–528. doi: 10.3892/etm.2013.1454, PMID: 24520240 PMC3919911

[ref54] Jiang W. Li M. He F. Zhou S. Zhu L. (2017). Targeting the NLRP3 inflammasome to attenuate spinal cord injury in mice. J. Neuroinflammation 14:207. doi: 10.1186/s12974-017-0980-9, PMID: 29070054 PMC5657095

[ref55] Jiang Z. Zhang J. (2021). Mesenchymal stem cell-derived exosomes containing miR-145-5p reduce inflammation in spinal cord injury by regulating the TLR4/NF-κB signaling pathway. Cell Cycle 20, 993–1009. doi: 10.1080/15384101.2021.1919825, PMID: 33945431 PMC8172161

[ref56] Kasravi M. Ahmadi A. Babajani A. Mazloomnejad R. Hatamnejad M. R. Shariatzadeh S.. (2023). Immunogenicity of decellularized extracellular matrix scaffolds: a bottleneck in tissue engineering and regenerative medicine. Biomaterials Research 27:10. doi: 10.1186/s40824-023-00348-z, PMID: 36759929 PMC9912640

[ref57] Kwon J. S. Kim G. H. Kim D. Y. Yoon S. M. Seo H. W. Kim J. H.. (2012). Chitosan-based hydrogels to induce neuronal differentiation of rat muscle-derived stem cells. Int. J. Biol. Macromol. 51, 974–979. doi: 10.1016/j.ijbiomac.2012.08.007, PMID: 22922106

[ref58] Lankford K. L. Arroyo E. J. Nazimek K. Bryniarski K. Askenase P. W. Kocsis J. D. (2018). Intravenously delivered mesenchymal stem cell-derived exosomes target M2-type macrophages in the injured spinal cord. PLoS One 13:e0190358. doi: 10.1371/journal.pone.0190358, PMID: 29293592 PMC5749801

[ref59] Li Q. Fu X. Kou Y. Han N. (2023). Engineering strategies and optimized delivery of exosomes for theranostic application in nerve tissue. Theranostics 13, 4266–4286. doi: 10.7150/thno.84971, PMID: 37554270 PMC10405842

[ref60] Li J. Mooney D. J. (2016). Designing hydrogels for controlled drug delivery. Nat Rev Mater 1:16071. doi: 10.1038/natrevmats.2016.71, PMID: 29657852 PMC5898614

[ref61] Li L. Mu J. Zhang Y. Zhang C. Ma T. Chen L.. (2022). Stimulation by exosomes from hypoxia preconditioned human umbilical vein endothelial cells facilitates mesenchymal stem cells Angiogenic function for spinal cord repair. ACS Nano 16, 10811–10823. doi: 10.1021/acsnano.2c02898, PMID: 35786851

[ref62] Li C. Qin T. Jin Y. Hu J. Yuan F. Cao Y.. (2023). Cerebrospinal fluid-derived extracellular vesicles after spinal cord injury promote vascular regeneration via PI3K/AKT signaling pathway. Journal of Orthopaedic Translation 39, 124–134. doi: 10.1016/j.jot.2023.02.001, PMID: 36909861 PMC9999163

[ref63] Li Y. Wang M. Sun M. Wang X. Pei D. Lei B.. (2022). Engineering antioxidant poly (citrate-gallic acid)-exosome hybrid hydrogel with microglia immunoregulation for traumatic brain injury-post neuro-restoration. Compos. Part B 242:110034. doi: 10.1016/j.compositesb.2022.110034

[ref64] Li L. Zhang Y. Mu J. Chen J. Zhang C. Cao H.. (2020). Transplantation of human mesenchymal stem-cell-derived exosomes immobilized in an adhesive hydrogel for effective treatment of spinal cord injury. Nano Lett. 20, 4298–4305. doi: 10.1021/acs.nanolett.0c00929 32379461

[ref65] Liang Y. Wu J. H. Zhu J. H. Yang H. (2022). Exosomes secreted by hypoxia-pre-conditioned adipose-derived mesenchymal stem cells reduce neuronal apoptosis in rats with spinal cord injury. J. Neurotrauma 39, 701–714. doi: 10.1089/neu.2021.0290, PMID: 35018814

[ref66] Lima R. Monteiro A. Salgado A. J. Monteiro S. Silva N. A. (2022). Pathophysiology and therapeutic approaches for spinal cord injury. Int. J. Mol. Sci. 23:13833. doi: 10.3390/ijms232213833, PMID: 36430308 PMC9698625

[ref67] Liu Z. Guo S. Dong L. Wu P. Li K. Li X.. (2022). A tannic acid doped hydrogel with small extracellular vesicles derived from mesenchymal stem cells promotes spinal cord repair by regulating reactive oxygen species microenvironment. Mater Today Bio 16:100425. doi: 10.1016/j.mtbio.2022.100425, PMID: 36186847 PMC9523385

[ref68] Liu W. Rong Y. Wang J. Zhou Z. Ge X. Ji C.. (2020). Exosome-shuttled miR-216a-5p from hypoxic preconditioned mesenchymal stem cells repair traumatic spinal cord injury by shifting microglial M1/M2 polarization. J. Neuroinflammation 17:47. doi: 10.1186/s12974-020-1726-7, PMID: 32019561 PMC7001326

[ref69] Liu S. Schackel T. Weidner N. Puttagunta R. (2017). Biomaterial-supported cell transplantation treatments for spinal cord injury: challenges and perspectives. Front. Cell. Neurosci. 11:430. doi: 10.3389/fncel.2017.00430 29375316 PMC5768640

[ref70] Liu W. Tang P. Wang J. Ye W. Ge X. Rong Y.. (2021). Extracellular vesicles derived from melatonin-preconditioned mesenchymal stem cells containing USP29 repair traumatic spinal cord injury by stabilizing NRF2. J. Pineal Res. 71:e12769. doi: 10.1111/jpi.12769, PMID: 34562326

[ref71] Liu Z. Tong H. Li J. Wang L. Fan X. Song H.. (2022). Low-stiffness hydrogels promote peripheral nerve regeneration through the rapid release of exosomes. Front. Bioeng. Biotechnol. 10:10. doi: 10.3389/fbioe.2022.922570 PMC9260118 35814007

[ref72] Liu X. Wu C. Zhang Y. Chen S. Ding J. Chen Z.. (2023). Hyaluronan-based hydrogel integrating exosomes for traumatic brain injury repair by promoting angiogenesis and neurogenesis. Carbohydr. Polym. 306:120578. doi: 10.1016/j.carbpol.2023.120578, PMID: 36746568

[ref73] Lu Y.-L. Dong S. He H. Li J. Wang X. Zhao H.. (2019). Enhanced photocatalytic activity of single-layered Hittorf’s violet phosphorene by isoelectronic doping and mechanical strain: a first-principles research. Comput. Mater. Sci. 163, 209–217. doi: 10.1016/j.commatsci.2019.03.042

[ref74] Lu Y. Zhou Y. Zhang R. Wen L. Wu K. Li Y.. (2019). Bone mesenchymal stem cell-derived extracellular vesicles promote recovery following spinal cord injury via improvement of the integrity of the blood-spinal cord barrier. Front. Neurosci. 13:209. doi: 10.3389/fnins.2019.00209, PMID: 30914918 PMC6423165

[ref75] Luo Y. He Y. Z. Wang Y. F. Xu Y. X. Yang L. (2023). Adipose-derived mesenchymal stem cell exosomes ameliorate spinal cord injury in rats by activating the Nrf2/HO-1 pathway and regulating microglial polarization. Folia Neuropathol. 61, 326–335. doi: 10.5114/fn.2023.130455, PMID: 37818693

[ref76] Luo Z. Peng W. Xu Y. Xie Y. Liu Y. Lu H.. (2021). Exosomal OTULIN from M2 macrophages promotes the recovery of spinal cord injuries via stimulating Wnt/β-catenin pathway-mediated vascular regeneration. Acta Biomater. 136, 519–532. doi: 10.1016/j.actbio.2021.09.026, PMID: 34551329

[ref77] Luo Z. Wu F. Xue E. Huang L. Yan P. Pan X.. (2019). Hypoxia preconditioning promotes bone marrow mesenchymal stem cells survival by inducing HIF-1α in injured neuronal cells derived exosomes culture system. Cell Death Dis. 10:134. doi: 10.1038/s41419-019-1410-y, PMID: 30755595 PMC6372680

[ref78] Ma Y. Wang K. Pan J. Fan Z. Tian C. Deng X.. (2019). Induced neural progenitor cells abundantly secrete extracellular vesicles and promote the proliferation of neural progenitors via extracellular signal-regulated kinase pathways. Neurobiol. Dis. 124, 322–334. doi: 10.1016/j.nbd.2018.12.003, PMID: 30528256 PMC6450400

[ref79] Mano J. F. Silva G. A. Azevedo H. S. Malafaya P. B. Sousa R. A. Silva S. S.. (2007). Natural origin biodegradable systems in tissue engineering and regenerative medicine: present status and some moving trends. J. R. Soc. Interface 4, 999–1030. doi: 10.1098/rsif.2007.0220, PMID: 17412675 PMC2396201

[ref80] Mazloomnejad R. Babajani A. Kasravi M. Ahmadi A. Shariatzadeh S. Bahrami S.. (2023). Angiogenesis and re-endothelialization in decellularized scaffolds: recent advances and current challenges in tissue engineering. Front. Bioeng. Biotechnol. 11:1103727. doi: 10.3389/fbioe.2023.1103727, PMID: 36873356 PMC9978201

[ref81] Michel M. Goldman M. Peart R. Martinez M. Reddy R. Lucke-Wold B. (2021). Spinal cord injury: a review of current management considerations and emerging treatments. J Neurol Sci Res 2:14.36037050

[ref82] Moeinabadi-Bidgoli K. Rezaee M. Hossein-Khannazer N. Babajani A. Aghdaei H. A. Arki M. K.. (2023). Exosomes for angiogenesis induction in ischemic disorders. J. Cell. Mol. Med. 27, 763–787. doi: 10.1111/jcmm.17689, PMID: 36786037 PMC10003030

[ref83] Mohammed I. Ijaz S. Mokhtari T. Gholaminejhad M. Mahdavipour M. Jameie B.. (2020). Subventricular zone-derived extracellular vesicles promote functional recovery in rat model of spinal cord injury by inhibition of NLRP3 inflammasome complex formation. Metab. Brain Dis. 35, 809–818. doi: 10.1007/s11011-020-00563-w 32185593

[ref84] Mu J. Li L. Wu J. Huang T. Zhang Y. Cao J.. (2022). Hypoxia-stimulated mesenchymal stem cell-derived exosomes loaded by adhesive hydrogel for effective angiogenic treatment of spinal cord injury. Biomater. Sci. 10, 1803–1811. doi: 10.1039/D1BM01722E, PMID: 35234220

[ref85] Mu J. Wu J. Cao J. Ma T. Li L. Feng S.. (2021). Rapid and effective treatment of traumatic spinal cord injury using stem cell derived exosomes. Asian J Pharm Sci 16, 806–815. doi: 10.1016/j.ajps.2021.10.002, PMID: 35027955 PMC8739259

[ref86] Mukhamedshina Y. O. Akhmetzyanova E. R. Kostennikov A. A. Zakirova E. Y. Galieva L. R. Garanina E. E.. (2018). Adipose-derived mesenchymal stem cell application combined with fibrin matrix promotes structural and functional recovery following spinal cord injury in rats. Front. Pharmacol. 9:343. doi: 10.3389/fphar.2018.00343, PMID: 29692732 PMC5902567

[ref87] Nie H. Jiang Z. (2021). Bone mesenchymal stem cell-derived extracellular vesicles deliver microRNA-23b to alleviate spinal cord injury by targeting toll-like receptor TLR4 and inhibiting NF-κB pathway activation. Bioengineered 12, 8157–8172. doi: 10.1080/21655979.2021.1977562, PMID: 34663169 PMC8806461

[ref88] Novikova L. N. Brohlin M. Kingham P. J. Novikov L. N. Wiberg M. (2011). Neuroprotective and growth-promoting effects of bone marrow stromal cells after cervical spinal cord injury in adult rats. Cytotherapy 13, 873–887. doi: 10.3109/14653249.2011.574116, PMID: 21521004

[ref89] Pan D. Zhu S. Zhang W. Wei Z. Yang F. Guo Z.. (2022). Autophagy induced by Schwann cell-derived exosomes promotes recovery after spinal cord injury in rats. Biotechnol. Lett. 44, 129–142. doi: 10.1007/s10529-021-03198-8, PMID: 34738222 PMC8854309

[ref90] Perale G. Rossi F. Sundstrom E. Bacchiega S. Masi M. Forloni G.. (2011). Hydrogels in spinal cord injury repair strategies. ACS Chem. Neurosci. 2, 336–345. doi: 10.1021/cn200030w, PMID: 22816020 PMC3369745

[ref91] Poongodi R. Chen Y. L. Yang T. H. Huang Y. H. Yang K. D. Lin H. C.. (2021). Bio-scaffolds as cell or exosome carriers for nerve injury repair. Int. J. Mol. Sci. 22:3347. doi: 10.3390/ijms222413347, PMID: 34948144 PMC8707664

[ref92] Pulido-Escribano V. Torrecillas-Baena B. Camacho-Cardenosa M. Dorado G. Gálvez-Moreno M. Á. Casado-Díaz A. (2022). Role of hypoxia preconditioning in therapeutic potential of mesenchymal stem-cell-derived extracellular vesicles. World J Stem Cells 14, 453–472. doi: 10.4252/wjsc.v14.i7.453, PMID: 36157530 PMC9350626

[ref93] Rauch M. F. Hynes S. R. Bertram J. Redmond A. Robinson R. Williams C.. (2009). Engineering angiogenesis following spinal cord injury: a coculture of neural progenitor and endothelial cells in a degradable polymer implant leads to an increase in vessel density and formation of the blood–spinal cord barrier. Eur. J. Neurosci. 29, 132–145. doi: 10.1111/j.1460-9568.2008.06567.x, PMID: 19120441 PMC2764251

[ref94] Ren J. Zhu B. Gu G. Zhang W. Li J. Wang H.. (2023). Schwann cell-derived exosomes containing MFG-E8 modify macrophage/microglial polarization for attenuating inflammation via the SOCS3/STAT3 pathway after spinal cord injury. Cell Death Dis. 14:70. doi: 10.1038/s41419-023-05607-4, PMID: 36717543 PMC9887051

[ref95] Rezaee M. Mohammadi F. Keshavarzmotamed A. Yahyazadeh S. Vakili O. Milasi Y. E.. (2023). The landscape of exosomal non-coding RNAs in breast cancer drug resistance, focusing on underlying molecular mechanisms. Front. Pharmacol. 14:1152672. doi: 10.3389/fphar.2023.1152672, PMID: 37153758 PMC10154547

[ref96] Riau A. K. Ong H. S. Yam G. H. F. Mehta J. S. (2019). Sustained Delivery System for Stem Cell-Derived Exosomes. Front. Pharmacol. 10:1368. doi: 10.3389/fphar.2019.01368, PMID: 31798457 PMC6868085

[ref97] Riedelová-Reicheltová Z. Brynda E. Riedel T. (2016). Fibrin nanostructures for biomedical applications. Physiol. Res. 65, S263–s272. doi: 10.33549/physiolres.933428, PMID: 27762592

[ref98] Roh E. J. Kim D. S. Kim J. H. Lim C. S. Choi H. Kwon S. Y.. (2023). Multimodal therapy strategy based on a bioactive hydrogel for repair of spinal cord injury. Biomaterials 299:122160. doi: 10.1016/j.biomaterials.2023.122160, PMID: 37209541

[ref99] Romanelli P. Bieler L. Heimel P. Škokić S. Jakubecova D. Kreutzer C.. (2021). Enhancing functional recovery through Intralesional application of extracellular vesicles in a rat model of traumatic spinal cord injury. Front. Cell. Neurosci. 15:795008. doi: 10.3389/fncel.2021.795008 35046776 PMC8762366

[ref100] Romanelli P. Bieler L. Scharler C. Pachler K. Kreutzer C. Zaunmair P.. (2019). Extracellular vesicles can deliver anti-inflammatory and anti-scarring activities of mesenchymal stromal cells after spinal cord injury. Front. Neurol. 10:1225. doi: 10.3389/fneur.2019.01225, PMID: 31849808 PMC6896947

[ref101] Rong Y. Liu W. Lv C. Wang J. Luo Y. Jiang D.. (2019b). Neural stem cell small extracellular vesicle-based delivery of 14-3-3t reduces apoptosis and neuroinflammation following traumatic spinal cord injury by enhancing autophagy by targeting Beclin-1. Aging (Albany NY) 11, 7723–7745. doi: 10.18632/aging.102283, PMID: 31563124 PMC6782003

[ref102] Rong Y. Liu W. Wang J. Fan J. Luo Y. Li L.. (2019a). Neural stem cell-derived small extracellular vesicles attenuate apoptosis and neuroinflammation after traumatic spinal cord injury by activating autophagy. Cell Death Dis. 10:340. doi: 10.1038/s41419-019-1571-8, PMID: 31000697 PMC6472377

[ref103] Samanta S. Rajasingh S. Drosos N. Zhou Z. Dawn B. Rajasingh J. (2018). Exosomes: new molecular targets of diseases. Acta Pharmacol. Sin. 39, 501–513. doi: 10.1038/aps.2017.162, PMID: 29219950 PMC5888687

[ref104] Siebert J. R. Eade A. M. Osterhout D. J. (2015). Biomaterial approaches to enhancing neurorestoration after spinal cord injury: strategies for overcoming inherent biological obstacles. Biomed. Res. Int. 2015:752572. doi: 10.1155/2015/752572 26491685 PMC4600545

[ref105] Sun G. Li G. Li D. Huang W. Zhang R. Zhang H.. (2018). hucMSC derived exosomes promote functional recovery in spinal cord injury mice via attenuating inflammation. Mater. Sci. Eng. C Mater. Biol. Appl. 89, 194–204. doi: 10.1016/j.msec.2018.04.006, PMID: 29752089

[ref106] Sun X. C. Wang H. Ma X. Xia H. F. (2023). Application of human umbilical cord mesenchymal stem cells in rat spinal cord injury model. ASAIO J. 69, e256–e264. doi: 10.1097/MAT.0000000000001938, PMID: 37039820

[ref107] Sung S. E. Seo M. S. Kim Y. I. Kang K. K. Choi J. H. Lee S.. (2022). Human epidural AD-MSC exosomes improve function recovery after spinal cord injury in rats. Biomedicine 10:678. doi: 10.3390/biomedicines10030678, PMID: 35327480 PMC8945172

[ref108] Sweis R. Biller J. (2017). Systemic complications of spinal cord injury. Curr. Neurol. Neurosci. Rep. 17:8. doi: 10.1007/s11910-017-0715-4 28188542

[ref109] Tsintou M. Dalamagkas K. Moore T. L. Rathi Y. Kubicki M. Rosene D. L.. (2021). The use of hydrogel-delivered extracellular vesicles in recovery of motor function in stroke: a testable experimental hypothesis for clinical translation including behavioral and neuroimaging assessment approaches. Neural Regen. Res. 16, 605–613. doi: 10.4103/1673-5374.295269, PMID: 33063708 PMC8067932

[ref110] Wang W. Huang X. Lin W. Qiu Y. He Y. Yu J.. (2018). Hypoxic preconditioned bone mesenchymal stem cells ameliorate spinal cord injury in rats via improved survival and migration. Int. J. Mol. Med. 42, 2538–2550. doi: 10.3892/ijmm.2018.3810, PMID: 30106084 PMC6192716

[ref111] Wang X. Jiang C. Zhang Y. Chen Z. Fan H. Zhang Y.. (2022). The promoting effects of activated olfactory ensheathing cells on angiogenesis after spinal cord injury through the PI3K/Akt pathway. Cell Biosci. 12:23. doi: 10.1186/s13578-022-00765-y, PMID: 35246244 PMC8895872

[ref112] Wang L. Pei S. Han L. Guo B. Li Y. Duan R.. (2018). Mesenchymal stem cell-derived exosomes reduce A1 astrocytes via downregulation of phosphorylated NFκB P65 subunit in spinal cord injury. Cell. Physiol. Biochem. 50, 1535–1559. doi: 10.1159/000494652, PMID: 30376671

[ref113] Wang H. Tang Q. Lu Y. Chen C. Zhao Y. L. Xu T.. (2023). Berberine-loaded MSC-derived sEVs encapsulated in injectable GelMA hydrogel for spinal cord injury repair. Int. J. Pharm. 643:123283. doi: 10.1016/j.ijpharm.2023.123283, PMID: 37536642

[ref114] Wang C. Wang M. Xia K. Wang J. Cheng F. Shi K.. (2021). A bioactive injectable self-healing anti-inflammatory hydrogel with ultralong extracellular vesicles release synergistically enhances motor functional recovery of spinal cord injury. Bioact Mater 6, 2523–2534. doi: 10.1016/j.bioactmat.2021.01.029, PMID: 33615043 PMC7873581

[ref115] Wang J. Wei Q. Yang Y. Che M. Ma Y. Peng L.. (2023). Small extracellular vesicles derived from four dimensional-culture of mesenchymal stem cells induce alternatively activated macrophages by upregulating IGFBP2/EGFR to attenuate inflammation in the spinal cord injury of rats. Front. Bioeng. Biotechnol. 11:1146981. doi: 10.3389/fbioe.2023.1146981, PMID: 37187882 PMC10176095

[ref116] Wu X. Wang L. Cong M. Shen M. He Q. Ding F.. (2020). Extracellular vesicles from skin precursor-derived Schwann cells promote axonal outgrowth and regeneration of motoneurons via Akt/mTOR/p70S6K pathway. Ann Transl Med 8:1640. doi: 10.21037/atm-20-5965, PMID: 33490152 PMC7812244

[ref117] Xiao Y. Hu X. Jiang P. Qi Z. (2023). Thermos-responsive hydrogel system encapsulated engineered exosomes attenuate inflammation and oxidative damage in acute spinal cord injury. Front. Bioeng. Biotechnol. 11:11. doi: 10.3389/fbioe.2023.1216878 PMC10442716 37614633

[ref118] Xiao X. Li W. Rong D. Xu Z. Zhang Z. Ye H.. (2021). Human umbilical cord mesenchymal stem cells-derived extracellular vesicles facilitate the repair of spinal cord injury via the miR-29b-3p/PTEN/Akt/mTOR axis. Cell Death Discovery 7:212. doi: 10.1038/s41420-021-00572-3, PMID: 34381025 PMC8357833

[ref119] Xie Y. Guan Q. Guo J. Chen Y. Yin Y. Han X. (2022). Hydrogels for exosome delivery in biomedical applications. Gels 8:328. doi: 10.3390/gels8060328, PMID: 35735672 PMC9223116

[ref120] Xie L. Wu H. Huang X. Yu T. (2023). Melatonin, a natural antioxidant therapy in spinal cord injury. Front. Cell Dev. Biol. 11:11. doi: 10.3389/fcell.2023.1218553 PMC10485268 37691830

[ref121] Xu S. Wang J. Zhong J. Shao M. Jiang J. Song J.. (2021). CD73 alleviates GSDMD-mediated microglia pyroptosis in spinal cord injury through PI3K/AKT/Foxo1 signaling. Clin. Transl. Med. 11:e269. doi: 10.1002/ctm2.269, PMID: 33463071 PMC7774461

[ref122] Yaghoobi A. Nazerian Y. Meymand A. Z. Ansari A. Nazerian A. Niknejad H. (2023). Hypoxia-sensitive miRNA regulation via CRISPR/dCas9 loaded in hybrid exosomes: a novel strategy to improve embryo implantation and prevent placental insufficiency during pregnancy. Frontiers in Cell and Developmental Biology 10:10. doi: 10.3389/fcell.2022.1082657 PMC9871368 36704201

[ref123] Yu Z. Li H. Xia P. Kong W. Chang Y. Fu C.. (2020). Application of fibrin-based hydrogels for nerve protection and regeneration after spinal cord injury. J. Biol. Eng. 14:22. doi: 10.1186/s13036-020-00244-3, PMID: 32774454 PMC7397605

[ref124] Zeng J. Gu C. Sun Y. Chen X. (2023). Engineering of M2 macrophages-derived exosomes via click chemistry for spinal cord injury repair. Adv. Healthc. Mater. 12:e2203391. doi: 10.1002/adhm.202203391, PMID: 36877863

[ref125] Zeng J. Gu C. Zhuang Y. Lin K. Xie Y. Chen X. (2023). Injectable hydrogel microspheres encapsulating extracellular vesicles derived from melatonin-stimulated NSCs promote neurogenesis and alleviate inflammation in spinal cord injury. Chem. Eng. J. 470:144121. doi: 10.1016/j.cej.2023.144121

[ref126] Zeng Y. Qiu Y. Jiang W. Shen J. Yao X. He X.. (2022). Biological features of extracellular vesicles and challenges. Front. Cell Dev. Biol. 10:816698. doi: 10.3389/fcell.2022.816698, PMID: 35813192 PMC9263222

[ref127] Zhai X. Chen K. Yang H. Li B. Zhou T. Wang H.. (2021). Extracellular vesicles derived from CD73 modified human umbilical cord mesenchymal stem cells ameliorate inflammation after spinal cord injury. J. Nanobiotechnol. 19:274. doi: 10.1186/s12951-021-01022-z, PMID: 34496892 PMC8425042

[ref128] Zhang X. Jiang W. Lu Y. Mao T. Gu Y. Ju D.. (2023). Exosomes combined with biomaterials in the treatment of spinal cord injury. Front. Bioeng. Biotechnol. 11:11. doi: 10.3389/fbioe.2023.1077825 PMC10040754 36994357

[ref129] Zhang B. Lin F. Dong J. Liu J. Ding Z. Xu J. (2021). Peripheral macrophage-derived exosomes promote repair after spinal cord injury by inducing local anti-inflammatory type microglial polarization via increasing autophagy. Int. J. Biol. Sci. 17, 1339–1352. doi: 10.7150/ijbs.54302, PMID: 33867850 PMC8040463

[ref130] Zhang N. Yin Y. Xu S. J. Wu Y. P. Chen W. S. (2012). Inflammation & apoptosis in spinal cord injury. Indian J. Med. Res. 135, 287–296. PMID: 22561613 PMC3361863

[ref131] Zhang Z. Zhang X. Wang C. Teng W. Xing H. Wang F.. (2022). Enhancement of motor functional recovery using immunomodulatory extracellular vesicles-loaded injectable thermosensitive hydrogel post spinal cord injury. Chem. Eng. J. 433:134465. doi: 10.1016/j.cej.2021.134465

[ref132] Zhang C. Zhang C. L. Xu Y. Li C. Cao Y. Li P. (2020). Exosomes derived from human placenta-derived mesenchymal stem cells improve neurologic function by promoting angiogenesis after spinal cord injury. Neurosci. Lett. 739:135399. doi: 10.1016/j.neulet.2020.135399, PMID: 32979457

[ref133] Zhang K. Zhao X. Chen X. Wei Y. du W. Wang Y.. (2018). Enhanced therapeutic effects of mesenchymal stem cell-derived exosomes with an injectable hydrogel for Hindlimb ischemia treatment. ACS Appl. Mater. Interfaces 10, 30081–30091. doi: 10.1021/acsami.8b08449, PMID: 30118197

[ref134] Zhao W. Tu H. Chen J. Wang J. Liu H. Zhang F.. (2023). Functionalized hydrogels in neural injury repairing. Front. Neurosci. 17:1199299. doi: 10.3389/fnins.2023.1199299, PMID: 37404462 PMC10315583

[ref135] Zheng X.-Q. Huang J. F. Lin J. L. Zhu Y. X. Wang M. Q. Guo M. L.. (2021). Controlled release of baricitinib from a thermos-responsive hydrogel system inhibits inflammation by suppressing JAK2/STAT3 pathway in acute spinal cord injury. Colloids Surf. B: Biointerfaces 199:111532. doi: 10.1016/j.colsurfb.2020.111532, PMID: 33385822

[ref136] Zhong D. Cao Y. Li C. J. Li M. Rong Z. J. Jiang L.. (2020). Neural stem cell-derived exosomes facilitate spinal cord functional recovery after injury by promoting angiogenesis. Exp. Biol. Med. (Maywood) 245, 54–65. doi: 10.1177/1535370219895491, PMID: 31903774 PMC6987743

[ref137] Zhou W. Silva M. Feng C. Zhao S. Liu L. Li S.. (2021). Exosomes derived from human placental mesenchymal stem cells enhanced the recovery of spinal cord injury by activating endogenous neurogenesis. Stem Cell Res Ther 12:174. doi: 10.1186/s13287-021-02248-2, PMID: 33712072 PMC7953814

[ref138] Zhou Y. Zhang X. L. Lu S. T. Zhang N. Y. Zhang H. J. Zhang J.. (2022). Human adipose-derived mesenchymal stem cells-derived exosomes encapsulated in pluronic F127 hydrogel promote wound healing and regeneration. Stem Cell Res Ther 13:407. doi: 10.1186/s13287-022-02980-3, PMID: 35941707 PMC9358082

[ref139] Zhou H.-L. Zhang X. J. Zhang M. Y. Yan Z. J. Xu Z. M. Xu R. X. (2016). Transplantation of human amniotic mesenchymal stem cells promotes functional recovery in a rat model of traumatic spinal cord injury. Neurochem. Res. 41, 2708–2718. doi: 10.1007/s11064-016-1987-9, PMID: 27351200

[ref140] Zhu X. Wang Z. Sun Y. E. Liu Y. Wu Z. Ma B.. (2021). Neuroprotective effects of human umbilical cord-derived mesenchymal stem cells from different donors on spinal cord injury in mice. Front. Cell. Neurosci. 15:768711. doi: 10.3389/fncel.2021.768711 35087378 PMC8787356

[ref141] Ziegler G. Grabher P. Thompson A. Altmann D. Hupp M. Ashburner J.. (2018). Progressive neurodegeneration following spinal cord injury: implications for clinical trials. Neurology 90, e1257–e1266. doi: 10.1212/WNL.0000000000005258, PMID: 29514946 PMC5890610

